# Supplementary material for: Selective knockout of PKA regulatory subunits reveal opposite catalytic and metabolic consequences with implications for Alzheimer’s disease
Source: bioRxiv. 2026 Jun 29:2026.06.26.734839. Preprint. [Version 1] doi: 10.64898/2026.06.26.734839 (PMC13345073; doi:10.64898/2026.06.26.734839)
Supplement: Supplement 2 [file NIHPP2026.06.26.734839v1-supplement-2.pdf]

bioRxiv preprint doi: https://doi.org/10.64898/2026.06.26.734839; this version posted June 29, 2026. The copyright holder for this preprint (which was not certified by peer review) is the author/funder, who has granted bioRxiv a license to display the preprint in perpetuity. It is made available under a CC-BY 4.0 International license.

SUPPLEMENTAL FIGURES

Figure S1

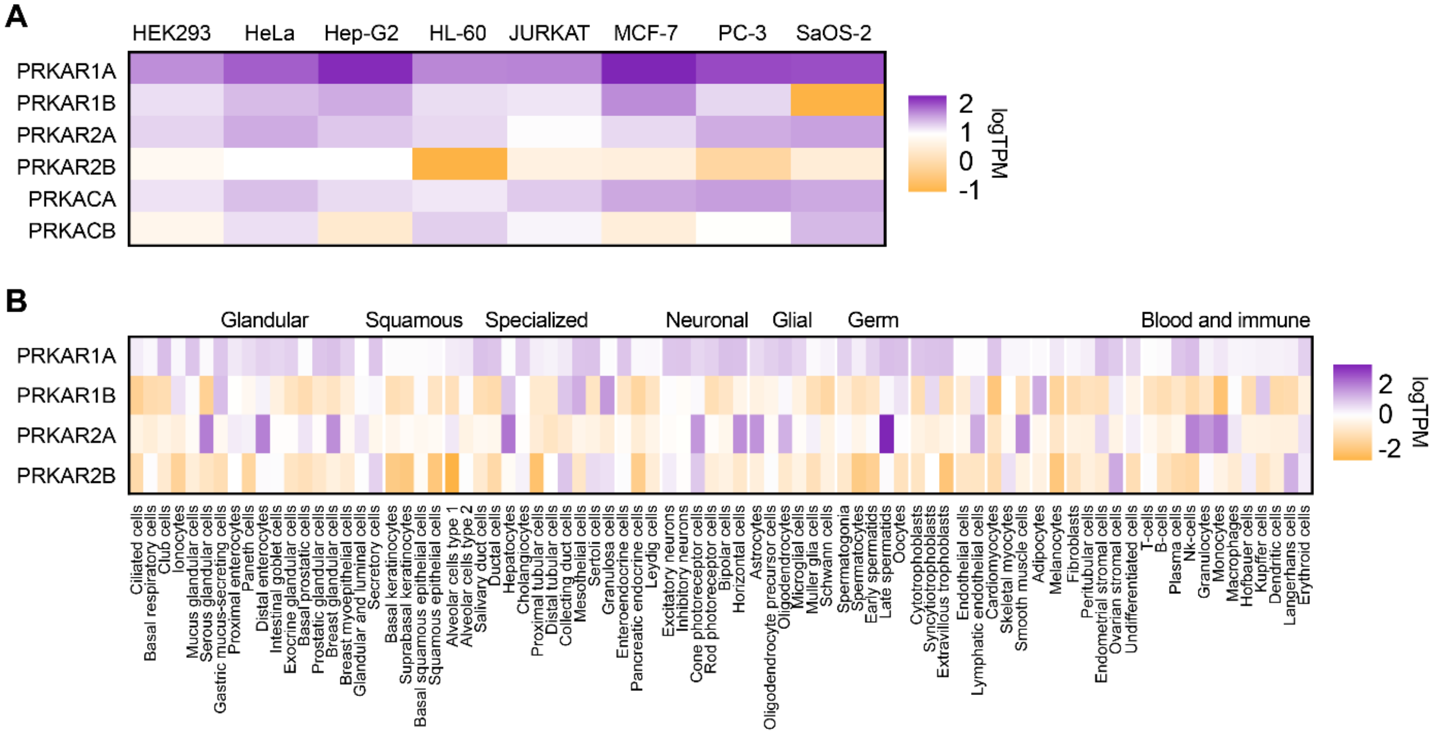

Figure S1. PKA subunit isoforms transcript counts across cell lines and types.

Transcript counts for PKA subunit isoforms across cell lines **(A)** and cell types **(B)**, from Jin, et al. (2023) and Karlsson, et al. (2021), respectively.

bioRxiv preprint doi: https://doi.org/10.64898/2026.06.26.734839; this version posted June 29, 2026. The copyright holder for this preprint (which was not certified by peer review) is the author/funder, who has granted bioRxiv a license to display the preprint in perpetuity. It is made available under a CC-BY 4.0 International license.

Figure S2

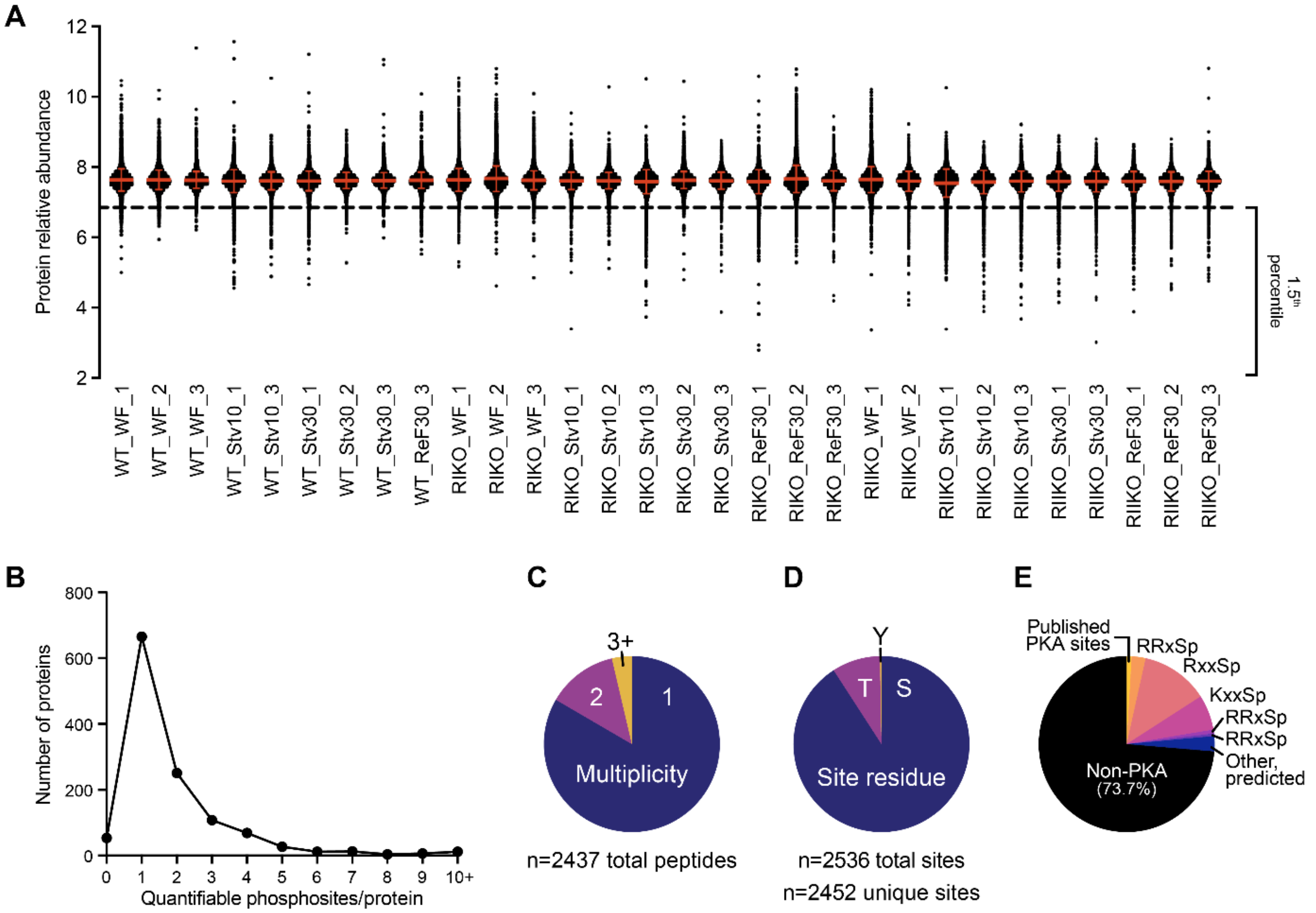

Figure S2. Supporting results from proteomics and phosphoproteomics study.

(A) Relative protein abundances for all detected proteins in the present study, showing no total proteome normalization differences across plexes, groups, and replicates. y=6.88 line represents the positive detection threshold for this study, set at the 1.5th percentile of all proteins detected.

(B) Histogram illustrating the distribution of proteins represented in our phosphoproteomics study. Some phosphopeptides were identified (MS2) but not quantified (MS3).

(C-E) Pie charts depicting proportions of phosphopeptides by feature detected in our study: multiplicity (C), residue type (D), and PKA motifs (E)

bioRxiv preprint doi: https://doi.org/10.64898/2026.06.26.734839; this version posted June 29, 2026. The copyright holder for this preprint (which was not certified by peer review) is the author/funder, who has granted bioRxiv a license to display the preprint in perpetuity. It is made available under a CC-BY 4.0 International license.

Figure S3

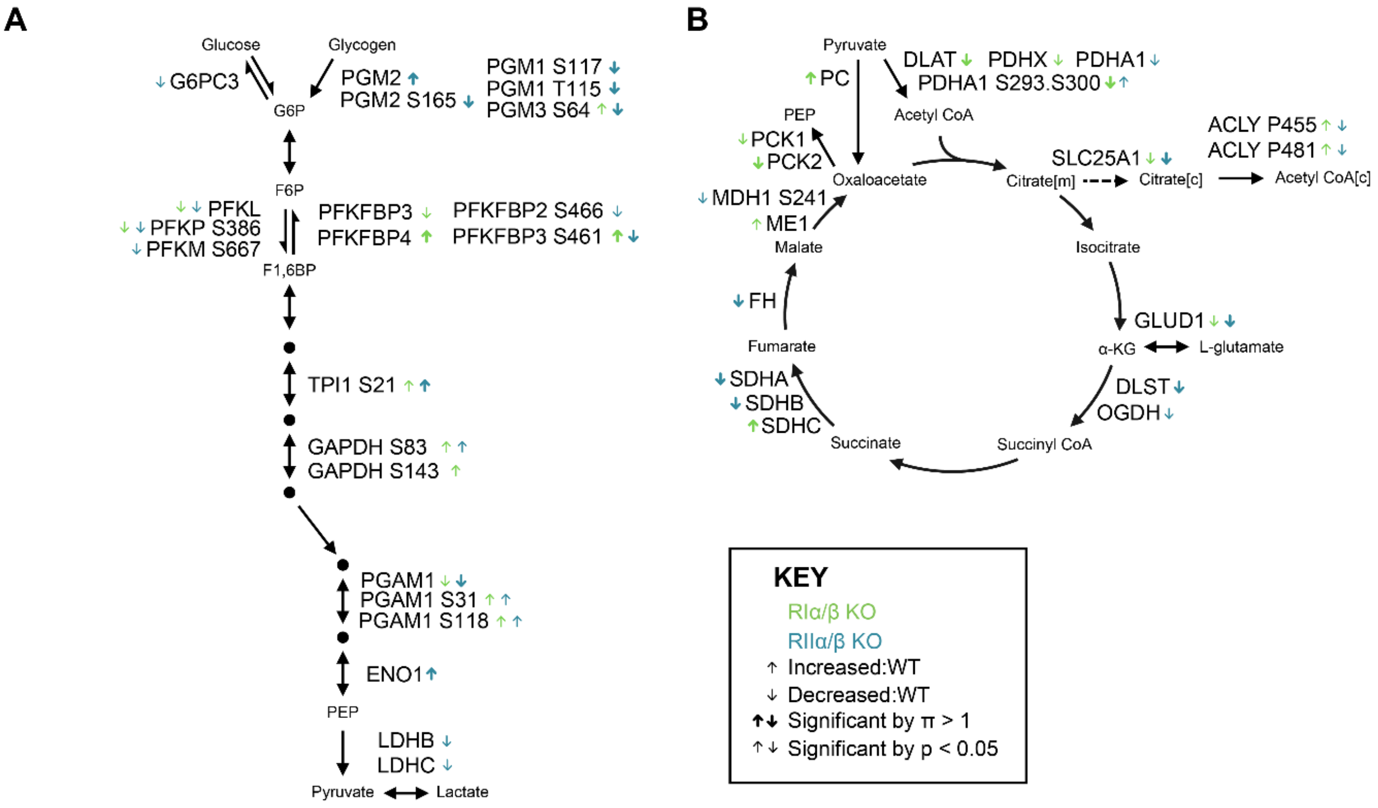

Figure S3. PKA RI and RII KO cells show distinct dysregulation of proteins and phosphosites regulating glucose metabolism.

Pathway schematics of glycolysis/gluconeogenesis **(A)** and the citric acid cycle **(B)**, with significant hits from either RI KO vs. WT or RII KO vs. WT annotated. Directionality of the change is indicated by arrows. P-values were calculated by limma. Significant hits were defined as $p < 0.05$ only (thin arrows) or both p-value (<0.05) and π-score (>1) (thick arrows). Proteins or phosphosites not annotated were either not significant for either comparison or not detected in the present study.

bioRxiv preprint doi: https://doi.org/10.64898/2026.06.26.734839; this version posted June 29, 2026. The copyright holder for this preprint (which was not certified by peer review) is the author/funder, who has granted bioRxiv a license to display the preprint in perpetuity. It is made available under a CC-BY 4.0 International license.

Figure S4

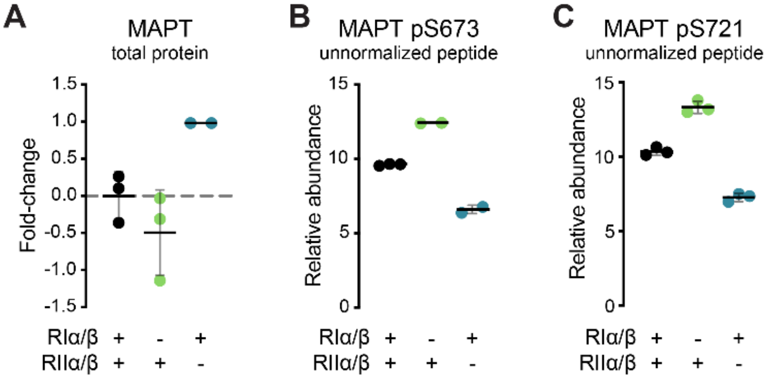

Figure S4. Corresponding to Fig. 5. Alterations in Tau phosphorylation are not due to total MAPT protein-level changes.

(A) MS quantification of MAPT total protein by KO cell line, relative to WT.

(B-C) MS quantification of MAPT phosphosite pS673 (B) and MAPT phosphosite pS721 (C), by KO cell line, relative to WT, corresponding to **Fig. 4A-B**. Raw peptide abundances not normalized to total protein level are represented. Data are represented as mean ± SD.

Figure S5

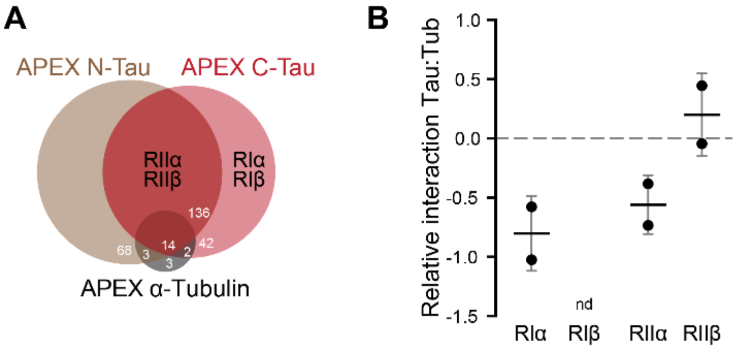

Figure S5. Corresponding to Fig. 5. APEX-Tau iPSC-neuron studies suggest strong Tau:RII interaction.

(A) Venn diagram illustrating RI vs. RII interactions with Tau and tubulin, from study Tracy, Madero-Pérez, and Swaney, et al. *Cell* (2022). Labeling was done in iPSC-neurons, quantification was done by label-free MS.

(B) R:Tau interaction relative to R:tubulin (Tub) from study Rossitto, et al. *BioRxiv* (2026). Labeling was done in iPSC-neurons, quantification was done by multiplexed MS.

bioRxiv preprint doi: https://doi.org/10.64898/2026.06.26.734839; this version posted June 29, 2026. The copyright holder for this preprint (which was not certified by peer review) is the author/funder, who has granted bioRxiv a license to display the preprint in perpetuity. It is made available under a CC-BY 4.0 International license.

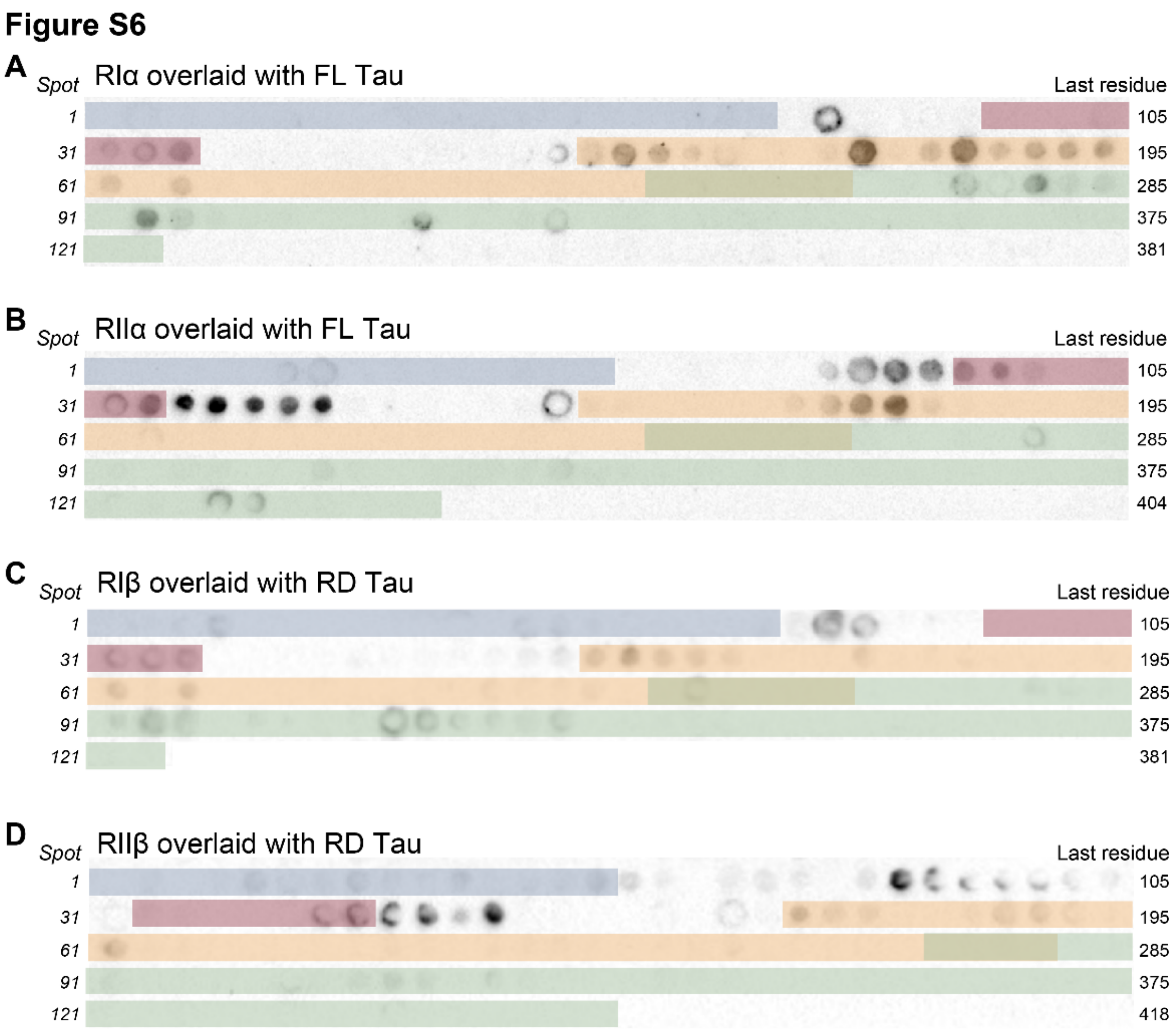

517 **Figure S6. Corresponding to Fig. 6. PKA-R:Tau peptide arrays show strong RII:RD Tau**
518 **interaction.**

519 **(A)** RIα peptide-binding array overlaid with FL Tau, quantified in **Fig. 5A**.

520 **(B)** RIIα peptide-binding array overlaid with FL Tau, quantified in **Fig. 5D**.

521 **(C)** RIβ peptide-binding array overlaid with RD Tau.

522 **(D)** RIIβ peptide-binding array overlaid with RD Tau.
